# Complete Vision-Based Traffic Sign Recognition Supported by an I2V Communication System

**DOI:** 10.3390/s120201148

**Published:** 2012-01-30

**Authors:** Miguel A. García-Garrido, Manuel Ocaña, David F. Llorca, Estefanía Arroyo, Jorge Pozuelo, Miguel Gavilán

**Affiliations:** 1 Electronics Department, Polytechnic School, University of Alcalá, Madrid 28871, Spain; E-Mails: mocana@depeca.uah.es (M.O.); estefania.arroyo@depeca.uah.es (E.A.); jorge.pozuelo@depeca.uah.es (J.P.); 2 Computer Engineering Department, Polytechnic School, University of Alcalá, Madrid 28871, Spain; E-Mails: llorca@aut.uah.es (D.F.L.); miguel.gavilan@aut.uah.es (M.G.)

**Keywords:** traffic sign recognition, advanced driver assistance systems, I2V, computer vision

## Abstract

This paper presents a complete traffic sign recognition system based on vision sensor onboard a moving vehicle which detects and recognizes up to one hundred of the most important road signs, including circular and triangular signs. A restricted Hough transform is used as detection method from the information extracted in contour images, while the proposed recognition system is based on Support Vector Machines (SVM). A novel solution to the problem of discarding detected signs that do not pertain to the host road is proposed. For that purpose infrastructure-to-vehicle (I2V) communication and a stereo vision sensor are used. Furthermore, the outputs provided by the vision sensor and the data supplied by the CAN Bus and a GPS sensor are combined to obtain the global position of the detected traffic signs, which is used to identify a traffic sign in the I2V communication. This paper presents plenty of tests in real driving conditions, both day and night, in which an average detection rate over 95% and an average recognition rate around 93% were obtained with an average runtime of 35 ms that allows real-time performance.

## 1. Introduction

The importance of safety for drivers, occupants and pedestrians has received an increasing interest recently, and traffic sign recognition (TSR) systems, within the Advanced Driving Assistance Systems (ADAS), play an important role, allowing to alert the drivers in potentially dangerous situations, for example when a driver may be speeding. Not only it is useful as a driving-assistance system, but TSR also has other possible applications, such as inventory system of traffic signs [1], or building and maintaining maps of signs. Furthermore it can be used as automatic inspection of signs. For example, VISUALISE system [2] provides automatic retro-reflection measurement of traffic signs and panels, allowing better maintained roads resulting in better and safer signposting.

This paper presents a complete traffic sign recognition system able to detect both circular and triangular signs and recognize up to one hundred of the main road signs. In outdoor detection, lighting conditions cannot be controlled. For this reason, analysis of the edges from grey-scale images have been used instead of colour-information that usually presents worst results under adverse lighting conditions. The classification stage is carried out by two Support Vector Machines (SVM) with Gaussian kernels. In detection stage circular and triangular road signs can be distinguished, so a SVM for circular signs and another SVM for triangular signs are applied. Finally, tracking of traffic signs is implemented using Kalman filtering techniques. For this purpose, a dynamic state model is defined, where measurements are obtained by means of a stereo pair of calibrated cameras.

One of the current open challenges in traffic sign recognition is to discard detected signs that do not pertain to the host road. The position of each detected traffic sign is obtained from stereo pair of cameras and those whose position are far from the vehicle lane will be discarded. However, there are some scenarios where the 3D relative position is not enough for discarding signs that do not apply to the host road. In those cases, information from infrastructure-to-vehicle (I2V) communication systems can be used as reliable solution. In [3,4] authors propose I2V systems based on RFID, but they have a short range and they need to determine the correct position of the tags over the road. We propose the use of wireless motes as support of the traffic sign recognition system. These systems are more flexible and help us to provide more precise information about the traffic sign and its position along the road by means of a GPS and a RF particle filter tracking system. This RF tracking system keeps the sign positioning even when GPS signal is lost.

The remainder of this paper is organized as follows: in Section 2 a revision of related works is done. Section 3 provides an overall description of the proposed vision-based sensor, focusing on the analysis of detection, classification, tracking and shows details of geometrical discarding of road signs. The I2V communication system is explained in Section 4, while experimental results that validate the proposed approach are presented and discussed in Section 5. Finally, Section 6 summarizes the conclusions and future work.

## 2. Related Work

A vision-based traffic sign recognition system can detect signs by their colour and shape. For that reason, in the literature we can find two main approaches to solve the problem of traffic sign recognition; segmentation, using colour-information, or analysis of the edges obtained from grey-scale images. When working with colour-information, relations between red-green-blue (RGB) components are used [5,6], but RGB colour-space is highly dependent on light, so other researchers work with hue-saturation-intensity (HSI), [1,7,8]. This colour-space is more immune to lighting changes. Thus in [7] two look-up tables (LUTs) for hue and saturation components are used to enhance red and blue colours, while in [1] a threshold over hue and saturation components is applied to find regions with high probability of having a traffic sign. However, the captured image is not completely invariant against changes in the chromaticity of the received light. The hue component changes with shades, climatic conditions or aging. Some works that improve the colour segmentation have been proposed. For example in [9] a human vision colour appearance model CIECAM97 is applied to extract colour information and to segment and classify traffic signs.

Among the works in which edge-analysis based on a grey-scale image is carried out, [10] must be highlighted, where Gavrila uses a template-based correlation method to identify potential traffic signs in images; this involves the so-called distance transforms (DT) in which starting from an edge-image, a matching with the template of those signs searched is carried out. These templates are organized hierarchically in order to reduce the number of operations. However, this method has a high computational cost for a real-time system. Another work to be remarked is the one developed by Barnes and Zelinsky [11] in which a variation of the Hough transform (HT) was used. The method used by Barnes and Zelinsky is based on [12], a fast method to detect points of interest using a system with radial symmetry. It uses the information of the magnitude and phase of the gradient of a grey-scale edge-image for different radii. Although the method is able to detect only circular signs, it has been extended to detect triangular, square and octagonal signs in [13]. A self-organizing map (SOM) has been used in [14] to extract contours in order to recognize shapes of traffic signs. Histograms of oriented gradient (HOG) have been used in [15] for pedestrian and road sign detection, which is suitable within boosting frameworks. Recently, new approaches in object detection have been applied. In [16] a set of colour-sensitive Haar wavelet features has been obtained from AdaBoost training and temporal information propagation, while in [17] a novel binary classifier through an evolutionary version of AdaBoost has been proposed.

The following stage is classification. The resultant image from the first stage is analysed by a classifier that determines whether the previously detected candidate regions are actual traffic signs or not. Neural networks, in their different topologies, are one of the most common tools employed [5,7,10]. A normalized image of the possible traffic signs is used in all cases as input vector. Although neural networks constitute the main tool used in the classification stage, it is not the only possibility. Another group of works use template-matching techniques. In [11] a normalized cross-correlation between the templates stored in database and the possible traffic signs is used, while in [18] a representation of road-sign data, based on extending the traditional normalized cross-correlation approach with a trainable similarity measure, is proposed. On the other hand, new approaches have been proposed where a SVM with Gaussian kernel is used to perform the classification stage [1,19].

The tracking stage provides memory to the system so that it takes into account not only a unique punctual instant for detection, but a whole sequence of images instead. Not all the works in this area include this feature, but those in which this approach is implemented reach better results. Among the latter, [1,6,16] may be highlighted. All of them make use of an extended Kalman filter [20] and use the 3D-position of the centre of the traffic sign as the state-vector.

Finally the use of wireless sensors in ITS applications is widespread nowadays. In [21] a study of some of these applications has been made. They are classified in: traffic safety, traffic law enforcement, traffic control, and smart parking. In [22] the collaboration between adjacent nodes is used to detect speed limit violations with high precision, within traffic law enforcement applications. We also have to highlight those works focusing on traffic safety and driver assistance system like [3], where an I2V communication and control system for intelligent speed control is proposed based upon Radio Frequency Identification (RFID) technology for identification of traffic signs on the road. Besides, in [23], a multisensor system is proposed that provides a red-light violation warning system for signalized intersections, and a speed advisory system for highways is applied.

In the context of the works discussed in this section our work provides solutions to the problems of coding contours, a set of classified signs larger than most of the works cited and a novel solution to the problem of discarding detected signs that do not pertain to the host road, which has not been addressed in any previous work.

## 3. Vision-Based Traffic Sign Recognition

### 3.1. Architecture Description

**Figure 1.**
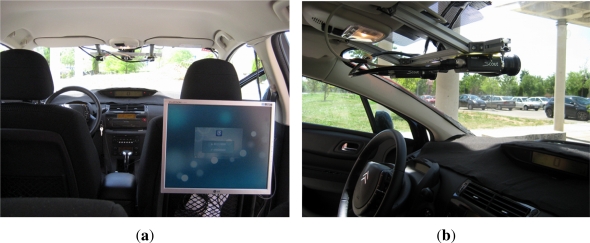
**(a)** Inside view of the experimental vehicle (a modified Citröen C4); **(b)** Stereo vision sensor.

The experimental vehicle used in this work is a modified Citröen C4 which can be seen in Figure 1. It has an onboard computer housing the image processing system, a differential GPS which is connected via RS232 serial port, a pair of synchronized low cost digital cameras connected via FireWire port and a wireless sensor 802.15.4 ZigBee Mote used as receiver in the I2V communication system. Besides, the CAN bus is used to connect engine control unit via serial port and thus obtain information from sensors, actuators, and other control devices.

This platform has been used previously in [24] as traffic data collection sensor for automotive applications, and in [25,26] as pedestrian detection sensor for collision avoidance applications. In this work the platform has been adapted for traffic sign recognition applications.

### 3.2. Detection

The aim of the first step in the image processing algorithm is to detect the precise location of the signs. In order to achieve this goal, an analysis of the obtained shapes from an edge-image is carried out. A Hough transform for straight lines is used to detect triangles, while a Hough transform for circles is applied to detect circular signs as well as the Stop sign, although the proposed method can also be used to detect rectangular or arrow signs, as shown in [2].

**Algorithm 1:** Pseudo code of encoding step algorithm.

1:	for *Y coord* =0, *Y coord* ¡ *height*, *Y coord* + + **do**
2:	**for***Xcoord* = 0, *Xcoord* ¡ *width*, *Xcoord* + + **do**
3:	*mode* = CLOCKWISE-SEARCH
4:	**while** CannyImage(*Xcoord*, *Y coord*) == WHITE **do**
5:	*ChangeDirectionRotation* = (VectorProduct(*previousV ector*, *actualV ector*) == CLOCKWISE) ? FALSE : TRUE
6:	**if***ChangeDirectionRotation* = TRUE **then**
7:	STOP
8:	**else**
9:	CONTINUE-SEARCHING-END-POINT
10:	**end if**
11:	**end while**
12:	*mode* = COUNTER-CLOCKWISE-SEARCH
13:	**while** CannyImage(*Xcoord*, *Y coord*) == WHITE **do**
14:	*ChangeDirectionRotation* = (VectorProduct(*previousV ector*, *actualV ector*) == COUNTER-CLOCKWISE) ? FALSE : TRUE
15:	**if***ChangeDirectionRotation* = TRUE **then**
16:	STOP
17:	**else**
18:	CONTINUE-SEARCHING-START-POINT
19:	**end if**
20:	**end while**
21:	**end for**
22:	**end for**
23:	**for** EACH *Contour_i_***do**
24:	**for** EACH *Contour_j_***do**
25:	**if***DirectionRotationContour_i_* = *DirectionRotationContour_j_***then**
26:	**if***EndPointContour_i_* NEAR *StartPointContour_j_***then**
27:	JoinContours(*Contour_i_*, *Contour_j_*)
28:	**end if**
29:	**end if**
30:	**end for**
31:	**end for**

**Figure 2.**
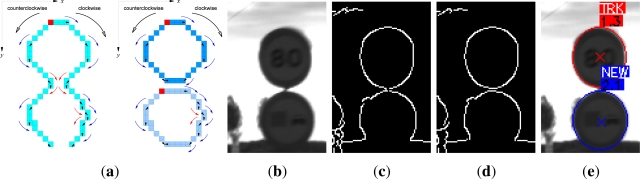
Encoding step, **(a)** graphical application of the algorithm; **(b)** Real sign; **(c)** Canny image; **(d)** split and merge; **(e)** Detection result.

The algorithm used to obtain edges is the Canny method. This method preserves contours, which is very important for detecting traffic signs using shape information because they are usually closed contours. With the aim of making the detection more reliable, we have chosen to adapt the two canny-thresholds in a dynamic way, depending on the histogram-distribution of the image. However, Canny algorithm presents three main problems when encoding contours: bifurcations, changes of direction and discontinuities. To solve these problems the Algorithm 1 is proposed, where the image is scanned from left to right and from top to bottom encoding the contours as a sequence of vectors. Circular and triangular signs are convex figures so the analysis of the vector product (direction of rotation) has to be the same for each new vector compared with the previous. If the direction got in the vector product changes, that means the contour tends to describe a figure of no interest, so this fact is used to split and merge contours as can be seen in Figure 2.

The contours are accepted if they are closed contours, or almost closed contours. In addition, they must also fulfill a certain aspect-ratio constraint. Actually, the Hough transform is only applied to accept contours after being filtered with this kind of restrictions. If all the contours in the image were analyzed the computational cost would be prohibitive, so all those contours that do not meet some requirements, typical of traffic signs, will be removed from the image, so that the computational time is reduced.

A straight line in the image plane can be defined in polar coordinates according to Equation (1), with a distance to the origin, *ρ*, and an angle between the normal line and the abscissa axis, *θ*.

(1) $$x_{i}\cdot \text{cos}(\theta )+y_{i}\cdot \text{sin}(\theta )=\rho$$

where the parameter space, *p* = (*ρ, θ*), must be quantized and expressed in a 2D accumulation matrix *a*, whose elements are initially set to zero. So, an element *a*(*ρ, θ*) is incremented by 1 for every contour point (*x_i_, y_i_*) in the image-domain, contained in the straight line with parameters (*ρ, θ*), as expressed in Equation (2), where a precision margin *ε* is introduced to compensate for quantization error when digitizing the image [27].

(2) $$|x_{i}\cdot \text{cos}(\theta_{t})+y_{i}\cdot \text{sin}(\theta_{t})=\rho_{r}|\;<\;ɛ$$

**Figure 3.**
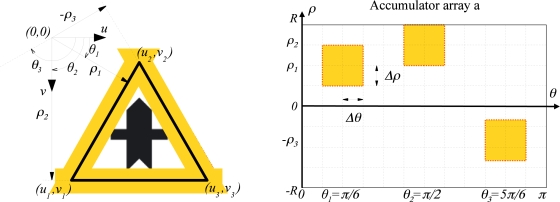
Constrained Hough transform applied to detect triangular, rectangular and arrow signs.

The aim is detecting three straight lines intersecting each other, forming a triangular sign. Different algorithms have been proposed in order to decrease the computational time of the Hough transform, e.g., a multi-dimensional quadtree structure for accumulating is suggested in [28] (coarse-to-fine method), or in [29] a method is proposed based on the fact that a single parameter space point can be determined uniquely with a pair, triple, or generally n-tuple of points from the original picture (many-to-one mapping method). In this work a constrained accumulation matrix *a* is proposed as a method to decrease the computational time, as it can be seen in Figure 3. The aim is to search for lines only in the shaded areas. The strategy is to apply the Hough transform to every contour, one after the other, hence every straight-line-parameters estimation is calculated by means of Equations (3) and (4), where (*x*_1_*, y*_1_) and (*x*_2_*, y*_2_) are points belonging to the contour under study.

(3) $$\rho =\frac{x_{1}\cdot y_{2}-x_{2}\cdot y_{1}}{\sqrt{{\left(x_{1}-x_{2}\right)}^{2}+{\left(y_{1}-y_{2}\right)}^{2}}}$$

(4) $$\theta =\arctan\frac{x_{1}-x_{2}}{y_{1}-y_{2}}$$

A similar strategy is followed for circular sign detection. Hough transform for circles is applied to detect circular signs and the stop sign too. A circumference in the image plane with center (*χ, ψ*) and radius *ρ* can be expressed as Equation (5).

(5) $${\left(x-\chi \right)}^{2}+{\left(y-\psi \right)}^{2}-\rho^{2}=0$$

where the parameter space *p* = (*χ, ψ, ρ*) must be quantized. For circumference detection the accumulator *a* will be a three-dimensional matrix with all elements initially set to 0. The element *a*(*χ, ψ, ρ*) is incremented by 1 for every contour point (*x_i_, y_i_*) in the image-domain, contained in the circumference with centre (*χ_r_, ψ_s_*) and radius *ρ_t_* as expressed in Equation (6), where a precision margin for the radius *ɛ* is introduced to compensate for quantization error when digitizing the image [27].

(6) $$|{\left(\chi_{r}-x_{i}\right)}^{2}+{\left(\psi_{s}-y_{i}\right)}^{2}-\rho_{t}^{2}|\;\;<ɛ$$

**Figure 4.**
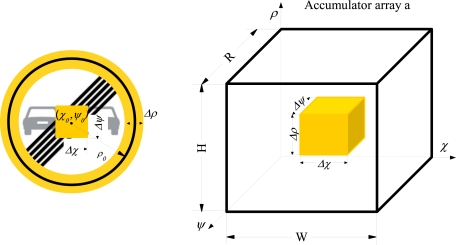
Constrained Hough transform applied to detect circular signs.

The circumference-parameters estimation is calculated using the direction of the contour-gradient under study, as in [12]. The search ranged into accumulator matrix *a* is constrained, the circumference-parameters are only searched inside shading areas, as it can be seen in Figure 4.

### 3.3. Recognition

**Figure 5.**
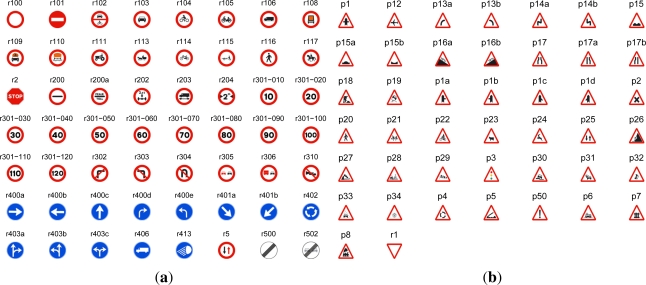
Classified set of traffic signs with their names in Spain, **(a)** circular signs, **(b)** triangular signs.

The selected candidates provided by the detection stage are classified by means of several SVM classifiers with Gaussian kernel and probability estimate output. In this work we can recognized up to one hundred of the most important Spanish traffic signs. Besides, we have exploited the fact that in the detection stage we can distinguish between circular and triangular signs, so a classifier for up to 56 circular signs, which are shown in Figure 5(a), and another classifier for up to 44 triangular signs, which are shown in Figure 5(b), are applied.

Although the SVM classifiers were designed to deal with binary classifications, it is straightforward to handle the multi-class case [30] by using the *one − against − one* classification algorithm. One of the main problems when working with hardly separable classes, in our case traffic signs, is the confusions between samples. In this paper we propose to group the classes into two hierarchical levels in both circular and triangular signs, so as to reduce the complexity of the problem and also the confusions between classes.

**Figure 6.**
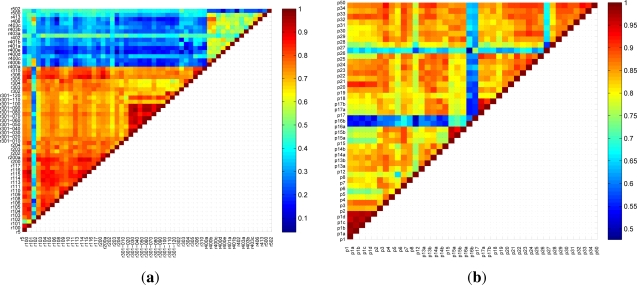
Matching results, **(a)** for circular signs, **(b)** for triangular signs.

We have obtained cross-correlation of all traffic signs in pairs based on Equation (7), proposed in [31], to measure the similarity between signs. Figure 6(a) shows the results of comparing each circular sign with the rest of signs, and the same for triangular signs (Figure 6(b)), where the axes represent the sign names. The color for each pair of signs measures their similarity. We have grouped those signs whose similarity are higher than 90%, (dark red), while the other signs are not grouped. Thus a grouped sign will be classified using two-level classifiers, and a non-grouped sign will have a direct classification, as shown in Table 1.

(7) $$R(x,y)=\frac{\sum_{x^{\prime },y^{\prime }}\left(T^{\prime }\left(x^{\prime },y^{\prime }\right)\cdot I^{\prime }\left(x+x^{\prime },y+y^{\prime }\right)\right)}{\sqrt{\sum_{x^{\prime },y^{\prime }}T^{\prime }{\left(x^{\prime },y^{\prime }\right)}^{2}\cdot \sum_{x^{\prime },y^{\prime }}I^{\prime }{\left(x+x^{\prime },y+y^{\prime }\right)}^{2}}}$$

**Figure 7.**
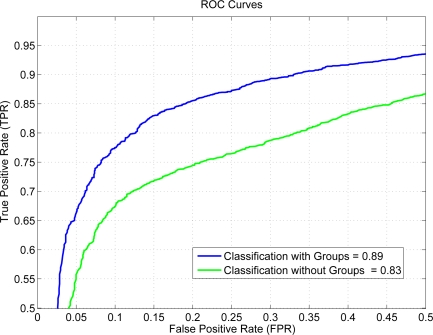
Signs recognition results, ROC curves.

Figure 7 depicts the ROC curves obtained for two classifiers, one without grouping signs (green) and the other one using the proposed groups in this work (blue), showing that the behavior of the proposed solution is better.

**Figure 8.**
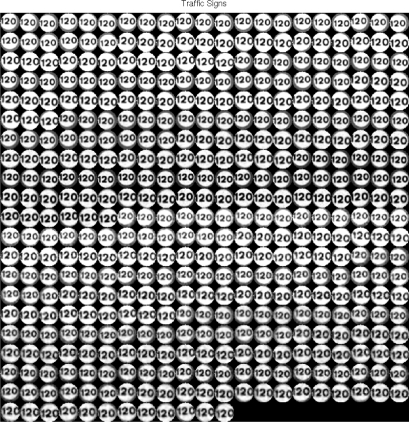
Transformations applied to a speed limit sign to generate the database.

The training database has been created from traffic sign pattern images applying transformations in order to simulate real conditions, such as rotations, noises, translations, grey-scale changes, *etc*. An example of application of this technique can be seen in Figure 8, where 432 transformations have been applied to a speed limit sign.

### 3.4. Tracking

After detecting consecutively an object classified as sign a predefined number of times (empirically set to 3 in this work), data association and tracking stages are triggered. Tracking is implemented using Kalman filtering techniques. For this purpose, a dynamic state model and a measurement model must be defined. The proposed dynamic state model is simple. Let us consider the state vector *x_n_*, defined as follows:

(8) $$x_{n}={\left[u,v,r,\dot{u},\dot{v},\dot{r}\right]}^{T}$$

In the state vector *u* and *v* are the respective horizontal and vertical image coordinates for the centre of every sign, and *r* is the radius in the image plane, while *u̇*, *v̇* and *ṙ* are their derivatives. A dynamical model equation can be written like this:

(9) $$x_{n+1}=A\cdot x_{n}+\omega_{n}$$

where *A* represents the system dynamics matrix and *ω* is the noise associated to the model. Although the definition of *A* is simple, it proves to be highly effective in practice. The model noise has been modelled as a function of distance and camera resolution. The state model equation is used for prediction in the first step of the Kalman filter. The next step is to define the measurement model. The measurement vector is defined as *z_n_* = [*u, v, r*]*^T^*. Then, the measurement model equation is established as follows:

(10) $$z_{n}=H\cdot x_{n}+v_{n}$$

In last equation *H* represents the measurement matrix and *υ_n_* is the noise associated to the measurement process.

**Figure 9.**
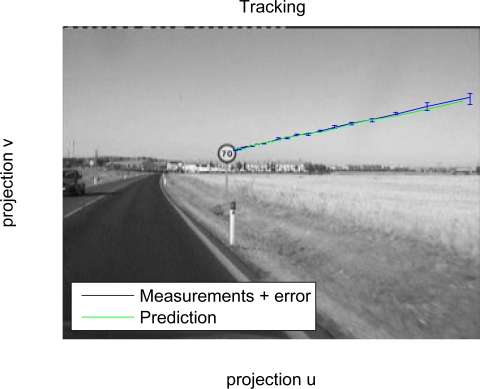
Measurements and prediction obtained during a tracking of a sign.

The tracking stage is triggered only if a sign is detected consecutively more than twice, and that sign will be tracked until it leaves the image. Thus, the purpose of the Kalman filtering is to obtain a more stable position of the detected signs during tracking, as shown in Figure 9.

### 3.5. Stereo Refinement

An accurate estimation of the relative position between the vehicle and the traffic sign has an important impact on further stages such as tracking and geometrical discarding. On top of that, it also helps to reduce the probability of considering the detected signs that do not pertain to the host road as pertaining to the host road. In order to minimize the error position, the relative distance is computed by using stereo vision. Monocular approaches have to apply some constraints such as flat terrain assumption and previous knowledge about the extensions or the height of the traffic sign, which introduce strong errors in the relative position estimate.

**Figure 10.**
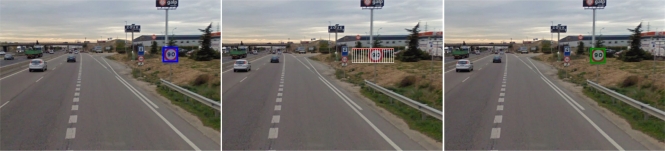
**(Left)** Left image and bounding box of the detected speed sign; **(Middle)** Search space along the epipolar line corresponding to the upper-left part of the bounding box on right image; **(Right)** Final match on right image.

**Figure 11.**
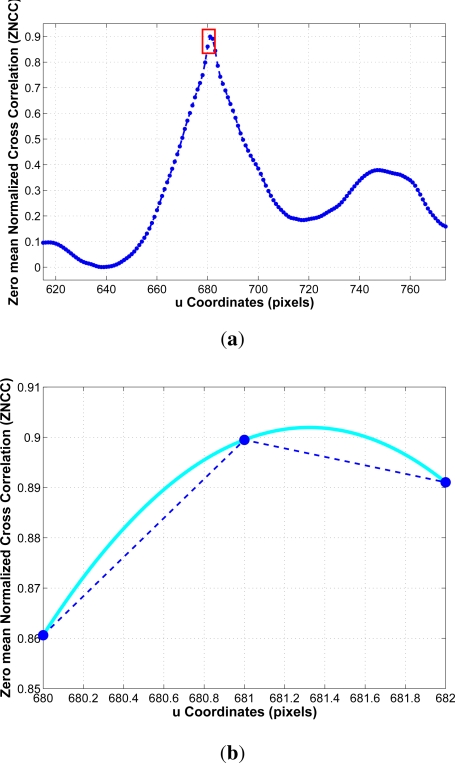
**(a)** Correlation function along the epipolar line; **(b)** Zoomed image around the global maximum and second degree polynomial approximation for getting subpixel accuracy.

Stereo parameters such as separation between cameras, sensor focal length and image resolution have been defined to reduce the error of the stereo measurements. As described in [25] we use a graphical method to define the sensor setup according to the application requirements. The calibration process is carried out on a supervised mode in order to minimize the calibration errors. Both images are undistorted and the epipolar geometry is computed. The content of the detected bounding boxes is matched along the epipolar line on the other stereo image by using the ZNCC function (Zero mean Normalized Cross Correlation) as in [32]. The search space can be reduced considering the minimum and maximum ranges. The correlation values are obtained and the values near the optimum are approximated by a second degree polynomial in order to compute the 3D position with subpixel accuracy [33]. This process is illustrated in Figures 10 and 11.

**Figure 12.**
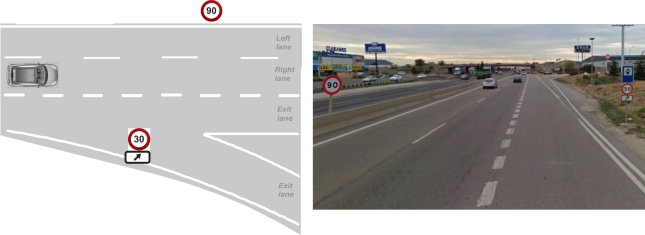
**(Left)** conflict scenario with two different speed signs; **(Right)** sample image.

Once we have the 3D relative position between the vehicle and the detected traffic signs, a simple discarding process can be applied in order to reject traffic signs that are located too far from the vehicle position (*i.e.*, traffic signs that belong to other roads). The thresholds used when discarding traffic signs are mainly defined according to several parameters such as type of road, number of lanes and expected road width. These parameters are computed using different hypothesis mainly related with the current lane of the host vehicle, and maximum distances allowed for both left and right traffic signs are finally obtained. However, there are some scenarios like the one depicted in Figure 12 where the 3D relative position is not enough for discarding signs that do not apply to the host road. Some of these cases can be extremely dangerous in the context of intelligent speed adaptation or assistance applications.

The vehicle global position is obtained from both the measurements provided by the GPS and the data supplied by the CAN bus (vehicle speed). However, the GPS and the CAN bus sample frequency is 1 Hz which implies that the system obtains one GPS measurement per each 20–30 m approximately (depending on the host speed). As the detection process is carried out at 28 Hz, we apply a linear interpolation between two consecutive GPS measurements. Finally, a global reference for each one of the detected signs is obtained by combining the global position of the vehicle and the relative position between the vehicle and the traffic sign. The main source of error when computing the relative distance between the traffic signs and the vehicle arises from the stereo vision, since as demonstrated in [25] for pedestrian detection, the maximum deviations coming from DGPS sensor are lower than 6 mm. The absolute and relative depth estimation errors are then determined by the stereo quantization error procedure as proposed in [25]. As described in a previous work [34] for obstacles detection, the mean relative depth error is below 3% for ranges up to 45 m. The global position of the traffic signs is very useful for inspection and inventory tasks, but we propose to also use this global position for solving scenarios like the one described in Figure 12 including I2V communications.

## 4. I2V Communications

**Figure 13.**
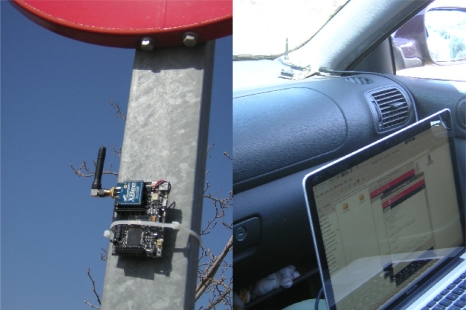
**(Left)** Waspmote transmitter installed on a signal. **(Right)** Waspmote receiver in the test-car.

In order to improve the traffic sign detection and solve the scenario of Figure 12, we propose an I2V communication system. The system is based on a low cost wireless transmitter that will be available in all the traffic signs. Actually, we are using a general purpose platform, Waspmote built by Libelium, and we have installed and set up several Waspmotes on traffic signs as transmitters. In addition, we have installed one of them as receiver in our test-car. Transmitter and receiver are shown in Figure 13.

Transmitter motes have been equipped with GPS receivers in order to send the global position of the signs to every car in the range of coverage. In addition, all the Waspmotes have been set up to transmit a message smaller than 94 bytes to avoid fragmentation with the following format and information at a frequency of 1 Hz, according to the GPS updating rate:

- Type of signal: max speed (X Km/h), stop, give way, *etc*. (2 bytes).
- Road name: useful to distinguish between main road and deceleration lane (70 bytes).
- Direction of the movement: increasing or decreasing (2 bytes).
- GPS: coordinates of global position (8 bytes for latitude and 8 bytes for longitude).

Waspmote platforms can be configured with several communication topologies (tree, mesh, peer to peer), but in any case, transmitter can always send a broadcast message. This characteristic has been exploited by our system to send the message of the traffic sign to all the cars that are in the coverage area (500 m in our case).

### 4.1. RF Mapping

Finally, in order to solve the GPS reception problems, we propose a RF mapping process that helps our process to maintain the traffic sign positioning even when the GPS signal has been lost.

The RF mapping process that we propose is based on our previous work [35]. It makes possible to estimate the position of the Waspmotes using the distance between them and the vehicle. First of all, the distance is obtained by means of using a path loss propagation model, and then the position of the signs are obtained using the distance and the knowledge of the car trajectory.

A particle filter [36] is used to achieve this aim, which is a sequential Monte Carlo algorithm, *i.e.*, a sampling method to approximate a distribution that uses its temporal structure. A “particle representation” of distributions is used in particular. We will be concerned with the distribution *P* (*X_bt_|z*_0:*t*_) where *X_bt_* = (*x_bt_, y_bt_, θ_bt_*) is the observed traffic sign at time *t*, and *z*_0:*t*_ = (*r*_1_*, r*_2_, …, *r_n_*) is the sequence of observations from time 0 to time *t*.

The transition and sensor models *P*(*X_bt_|z*_0:*t*_) are represented using a collection of *N* weighted samples or particles $\left\{X_{\mathrm{bt}}^{\left(i\right)},\;\pi_{t}^{\left(i\right)}\right\}\underset{i=1}{N}$ where $\pi_{t}^{\left(i\right)}$ is the weight of particle $X_{\mathrm{bt}}^{\left(i\right)}$ (Equation (11)).

(11) $$P\left(X_{\mathrm{bt}}|z_{0:t}\right)\approx \sum_{i}\pi_{t-1}\delta \left(X_{\mathrm{bt}}-X_{\mathrm{bt}-1}^{\left(i\right)}\right)$$

Firstly, the particles are uniformly distributed within a “ring” with radius equal to the first range measurement. We make this “ring” wide enough in order to absorb the signal noise.

Secondly, the particles are not propagated using any motion model since we know that the traffic signs are static. Instead, we apply a small random noise to the position of the particles in order to avoid that all the particles stay at the same position.

Finally, the particles are updated by the previous actions *a*_*t*−1_ and the actual observation *z_t_*. Finally, it is important to highlight that this algorithm does not need to collect a high number of samples to estimate the sign position, since it is an online process.

## 5. Experimental Results

The results presented in this paper were obtained from video sequences in real traffic performance under different lighting conditions, in sunny or cloudy days, in the rain and at night. We calculate the performance of the whole system over a test set of 60.000 stereo pairs of images with a 320 × 240 resolution, which correspond to 80 km road.

The results have been obtained taking into account that signs detected are considered positive samples (P), while negative samples (N) are noisy objects detected and not signs. Thus, every sign can be detected or classified as: true positive (TP) if the outcome from prediction and actual value are positive; however if the actual value is negative then a false positive (FP) is obtained. Conversely, a true negative (TN) occurs when both the prediction outcome and the actual value are negative. Finally, when the prediction outcome is negative and the actual value is positive a false negative (FN) is obtained.

The results obtained in the detection stage are summarized in the Table 2, where we can highlight the sensitivity obtained, over 95% in both circular and triangular signs, and the low number of false negative obtained, especially for triangular signs. In addition, the proposed system works in a very similar way in both day and night.

A tracked sign will be considered classified if the output of the classifier is greater than a certain threshold for more than five times. At night it is more difficult to classify the signs, especially the circular ones because they have less variability, as they are shown in the Table 3, where the sensitivity for circular signs at night reaches only 80%. However, both sensitivity and precision obtained for the other signs, under any lighting conditions, are higher than 92%.

**Figure 14.**
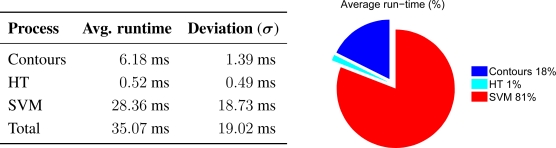
Average run-time for different phases of implementation, in frames with traffic signs.

One of the objectives of this work is to obtain a system capable of working in real time. In that sense we measured the average run-time, as depicted in Figure 14, of the following processes:

- Process of extracting, encoding and selecting contours, (*contours*).
- Process of Hough Transform implementation, (*HT*).
- Process of tracking and classification, (*SVM*).

Although all results have been obtained by means of an offline process, the average runtime obtained, 35 ms with 19 ms deviation, would allow real-time performance (The performance of system, under different lighting conditions, can be seen in the following videos: during day time http://www.youtube.com/v/a7E337WVbFc, with light rain http://www.youtube.com/v/3XKcZV-ztl8, and at night http://www.youtube.com/v/6BX-0ROa5lI). A real-time implementation is considered as an improvement to be realized in future work.

**Figure 15.**
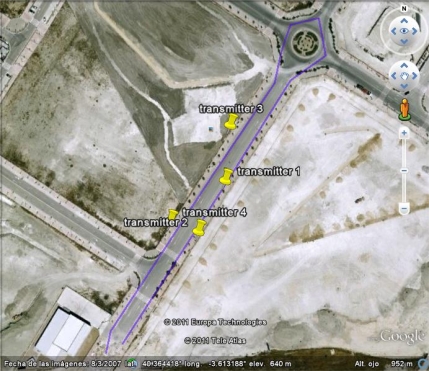
Test-bed. In violet color, the path of the car. In yellow, the four transmitters.

Finally, in order to check our I2V infrastructure, we have developed a test-bed that consists of four Waspmotes, configured as transmitters, installed in four traffic signs (see Figure 15), and one Waspmote, configured as receiver, installed in a car.

First experiment was conducted to check the maximum received packets per second from each transmitter independently. The car followed a path (marked as a straight line in violet color in Figure 15) at a 60 km/h. At a first time only transmitter 1 was configured to send messages at its maximum speed while the other three were switched off, and the Waspmote installed in the car received a maximum of 5 packets per second. This experiment was repeated for the other three transmitters. In all of the experiments, 5 packets were the maximum packets received.

**Figure 16.**
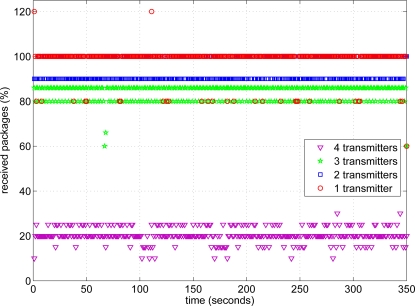
Receiving packets from several transmitters.

Once we knew that 5 packets per second was the maximum speed of transmission, we configured all transmitters to send 5 messages per second. Then, we checked what was the effect of having several transmitters sending information about the signs at the same time. Firstly, we switched on only one transmitter and we evaluated the received rate along several trips of the car (more than five minutes). Then, we activated two transmitters at the same time and the experiment was repeated, and so on for the four transmitters. Figure 16 shows the results of this experiment.

As it can be seen in Figure 16, when only one transmitter is sending information almost all the packets are received. Sometimes, more than five packets are received (giving more than 100%), due to synchronization problems in the transmitting-receiving process. When two or three transmitters are sending at the same time, the rate can be reduced down to 80%, but in any case four packets are received from each transmitter. The main problem appeared when four transmitters were sending messages at the same time. In this case, the receiving rate fell down to 20% and even 0% when the receiver was blocked out. In this case, the main problem was related to the receiving buffer of the Waspmote. When it was filled out, receiver was blocked out and it was not able of processing more messages. Therefore, we decided to reduce the transmitting rate down to two or three packets per second according to the number of the traffic signs.

## 6. Conclusions

This paper presents a complete traffic sign recognition system that works under different light conditions, both day and night. An important aspect to be highlighted is that the detection algorithm is adaptive, this adaptation is achieved mainly due to two factors, first the use of adaptive thresholds applied to Canny algorithm to obtain contours, which change their values depending on the histogram function at any time, and second, the application of Hough transform depending on the information received from every candidate contour. In addition, we have proposed an algorithm to encoding contours that solves, in many cases, problems of bifurcations, changes of direction and discontinuities. So the experimental results in detection indicate that the proposed vision-based sensor is reliable and accurate since it has obtained an average detection rate over 95% in all lighting conditions, and also can be applied not only for circular or triangular signs but also to rectangular or arrow signs.

A set of up to one hundred different traffic signs have been classified, both circular and triangular. This large set of signs is an improvement regarding other works. In addition, the proposed training technique is based on: firstly, transformations applied to traffic sign pattern images to simulate real conditions, and secondly, grouping traffic signs according to their similarity. This has resulted in a high recognition rate, as shown in the Table 3, where the sensitivity and precision obtained is above 92% in all cases except for circular signs at night, where the sensitivity decreases up to 80% due to the high variability and lack of contrast of the signs.

A comparison of the performance of our proposal with other existing approaches will be very useful to clearly provide the baseline of the improvements obtained with our system. However, the lack of a common evaluation framework for traffic sign detection systems (public available datasets, labelled data, *etc*.) makes it impossible to perform such kind of comparison. We remark that this problem should be addressed in future research work.

Finally, the use of I2V communication system is a novel solution to the problem of discarding detected signs that do not pertain to the host road instead of using geometrical constraint. Installation of Waspmote sensors on all traffic signs is both technically and economically unviable. However, these sensors can be installed in those areas where there may be confusion between signs, such as at intersections, entrance or exit ramps on highways. As revealed in our experiments, the best solution is to use a low number of sensors as transmitters. Therefore, in the near future we have the intention of reducing the number of sensors per sign by means of using them only in conflictive areas and providing information of all the signs in that area. With this, we hope to reduce the cost and to avoid packet loss.

For future work, the use of the vehicle dynamics (vehicle direction, trajectory, yaw rate, steering wheel position, speed changes, *etc*.) can be considered to increase the robustness of the traffic sign discarding process. The automatic traffic sign recognition system is useful as a driving-assistance system, but it also has other possible applications, such as inventory system of traffic signs, building and maintaining maps of signs, or automatic inspection of signs in order to providing a better maintenance and safer signposting. All these applications involve a challenging research work for the near future.

## Figures and Tables

**Table 1.** Signs and groups classified.

**Type**	**total signs**	**groups**	**grouped signs**	**non grouped signs**
Circular	56	7	27	29
Triangular	44	5	24	20

**Table 2.** Summary of detection results under different lighting conditions.

**Classes**	**Light**	**Signs**	**TP**	**FN**	**FP**	**sensitivity**	**precision**
Circular	Day	143	136	7	52	95.10%	72.34%
	Night	64	61	3	20	95.31%	75.31%
Triangular	Day	87	86	1	12	98.86%	87.75%
	Night	46	44	2	0	95.65%	100.00%

**Table 3.** Summary of recognition results under different lighting conditions.

**Signs**	**Light**	**TP**	**FN**	**FP**	**TN**	**sensitivity**	**precision**
Circular	Day	127	9	4	48	93.38%	92.31%
	Night	49	12	1	19	80.33%	95.00%
Triangular	Day	84	2	6	6	97.67%	93.33%
	Night	44	0	0	0	100.00%	100.00%
